# Analysis of Covalently Bound Microcystins in Sediments and Clam Tissue in the Sacramento–San Joaquin River Delta, California, USA

**DOI:** 10.3390/toxins12030178

**Published:** 2020-03-13

**Authors:** Melissa Bolotaolo, Tomofumi Kurobe, Birgit Puschner, Bruce G Hammock, Matt J. Hengel, Sarah Lesmeister, Swee J. Teh

**Affiliations:** 1 Department of Anatomy, Physiology, and Cell Biology, University of California, Davis, CA 95616, USA; tkurobe@ucdavis.edu (T.K.); brucehammock@gmail.com (B.G.H.); sjteh@ucdavis.edu (S.J.T.); 2 Department of Molecular Biosciences, University of California, Davis, CA 95616, USA; puschner@msu.edu; 3 College of Veterinary Medicine, Michigan State University, East Lansing, MI 48824, USA; 4 Department of Environmental Toxicology, University of California, Davis, CA 95616, USA; mjhengel@ucdavis.edu; 5 California Department of Water Resources, West Sacramento, CA 95814, USA; Sarah.Lesmeister@water.ca.gov

**Keywords:** cyanotoxins, microcystins, harmful algal blooms, covalently bound, sediment, clam tissues, chemical analysis, GC–MS

## Abstract

Harmful cyanobacterial blooms compromise human and environmental health, mainly due to the cyanotoxins they often produce. Microcystins (MCs) are the most commonly measured group of cyanotoxins and are hepatotoxic, neurotoxic, and cytotoxic. Due to MCs ability to covalently bind to proteins, quantification in complex matrices is difficult. To analyze bound and unbound MCs, analytical methods were optimized for analysis in sediment and clam tissues. A clean up step was incorporated to remove lipids, improving percent yield. This method was then applied to sediment and clam samples collected from the Sacramento–San Joaquin River Delta (Delta) in the spring and fall of 2017. Water samples were also tested for intracellular and extracellular MCs. These analyses were used to quantify the partitioning of MCs among sediment, clams, and water, and to examine whether MCs persist during non-summer months. Toxin analysis revealed that multiple sediment samples collected in the Delta were positive for MCs, with a majority of the positive samples from sites in the San Joaquin River, even while water samples from the same location were below detection limit. These data highlight the importance of analyzing MCs in complex matrices to accurately evaluate environmental risk.

## 1. Introduction

Harmful cyanobacterial blooms (cyanoHABs) occur when proliferated growth of cyanobacterial species cause adverse effects in aquatic environments, compromising both environmental and human health. These effects may include fish kills via depletion of oxygen, reduction of beneficial phytoplankton by competition for nutrients, and reduction of water quality through production of odorous and toxic compounds [1,2]. A major threat of cyanoHABs is the cyanotoxins produced by some cyanobacterial species, which have been implicated in the deaths of livestock and companion animals as well as liver damage in humans [3,4,5,6]. The most frequently detected class of cyanotoxins is microcystins (MCs). CyanoHABs and their associated MCs contaminate waters worldwide such as Lake Taihu in China and Lake Erie in the US, compromising drinking water supplies for millions of people [7,8]. MC intoxication in humans has also been reported worldwide. Chinese fisherman who regularly ingest fish in MC-contaminated waters had significant levels of MCs in their serum as well as biochemical indicators of liver damage [9]. In Caruaru, Brazil, over fifty people died after being inadvertently exposed when water contaminated with MCs was used for hemodialysis [10]. Eutrophication and warmer water temperatures encourage growth of toxic cyanobacteria over non-toxic species. Therefore, the cyanoHABs will likely worsen with predicted rises in global temperatures and eutrophication in coming years [11,12].

The toxicity of MCs is largely due to the toxin’s inhibition of protein phosphatases (PPs) and their covalent binding to proteins, a phenomenon which also contributes to MC bioaccumulation. The most common route of MC exposure is through ingestion, after which MCs can be actively transported into hepatocytes where the toxins interact with PPs [13]. Within minutes following contact, MCs reversibly interact with the active site of PPs; after a few hours of exposure, the bond between MCs and PPs becomes covalent and largely irreversible [14,15]. Both the reversible and covalent binding of MCs to PPs inhibit PP activity which leads to hyperphosphorylation, cytoskeleton reconstruction, cell death, and ultimately liver damage [16,17]. MCs have been found to not only bind to PPs, but also to other proteins that contain cysteine groups [18]. This covalent binding causes MCs to bind to proteins and accumulate in animal tissue, leading to bioconcentration, bioaccumulation, and possibly biomagnification [19,20,21]. Bioaccumulation of MCs is a public health concern as multiple studies have found significant concentrations of MCs in tissues of fish above the World Health Organization’s suggested tolerable daily intake (TDI) for humans [22,23]. 

Monitoring for MCs in the environment generally targets the water column; however, MCs have also been shown to accumulate in the benthic environment [13,14,15,16]. MCs in the benthos can originate from a number of sources. One potential route is the biomass from cyanoHABs and associated MCs that sink to the sediment following a bloom. Recent studies have also documented a number of benthic cyanobacteria capable of producing MCs [24]. In addition, the bottom of the water column is typically a cooler environment with attenuated sunlight, slowing the rate of MC degradation via abiotic processes [25,26]. It is argued that sediments may serve as an important elimination route for MCs in the environment; however, bound MCs are still able to induce toxicity [27]. In an exposure study with mice, MC conjugates were found to have approximately one-third to one-tenth of the toxicity of free MCs [18]. Bound MCs also can be cleaved enzymatically, becoming free and bioavailable, and therefore covalently bound MCs in the benthos have potential to be distributed throughout the food web [28,29]. MCs have been found to accumulate in mussels, fish, clams, and zooplankton [30,31,32,33]. Sunfish fed with MC-rich zooplankton bioaccumulated MCs, demonstrating potential for biomagnification [34].

**Figure 1:**
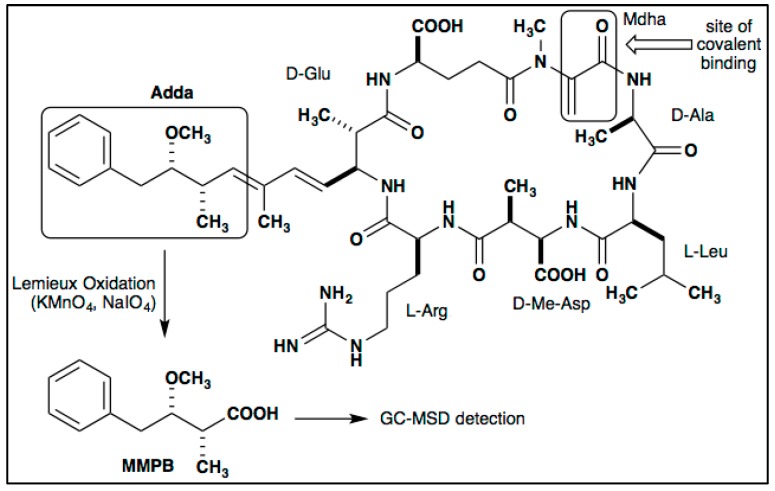
Structure of MC-LR and yield of 3-methyl-2-methoxy-phenylbutyric acid (MMPB) via oxidative cleavage.

Depending on the form of MCs, free or covalently bound, there are different types of methods used to detect MCs. MCs are typically extracted from water samples by lysing the cyanobacterial cells and extracting MCs with a solvent such as methanol. The extract may be analyzed via multiple analytical methods including enzyme-linked immunosorbent assay (ELISA), protein phosphatase inhibition assay (PPIA), and liquid chromatography–mass spectroscopy (LC–MS). However, with solvent extraction, the bound fraction of MCs is often not accurately measured by PPIA, LC–MS, or ELISA [19,35,36]. To accurately quantify bound MCs, an oxidant (usually in the form of potassium permanganate or ozone) is used to cleave the double bond on the Adda moiety, the 5th amino acid of the heptapeptide MC structure, releasing 3-methyl-2-methoxy-phenylbutyric acid (MMPB) [37] (Figure 1). MMPB can then be quantified using gas chromatography–mass spectroscopy (GC–MS) or LC–MS to indirectly determine the concentration of total MCs [38]. To date, there are more than 100 characterized MC congeners [39]. However, there are a limited number of commercially available MC congener standards and total MC concentration is often underestimated. The MMPB method identifies a moiety which is present on all MC congeners and is, therefore, the most effective method of estimating total MC concentration. Techniques used in biomonitoring of MCs commonly evaluate only the unbound fraction; therefore, total MC concentration could be drastically underestimated in many assessments. The covalently bound fraction of MCs often represents a large portion of MCs found in the environment. Bound MCs may have toxicities that are approximately an order of magnitude less than free MCs, yet they are sometimes detected at levels multiple orders of magnitudes more than free MCs [19]. For example, a report analyzing dungeness crab larvae, *Metacarcinus magister*, found the covalently bound concentration of MCs to be 10,000 times the concentration of unbound MCs [19]. 

Here, we examined the partitioning of bound and unbound MCs among sediment and clams as well as unbound MCs in water samples of the Sacramento–San Joaquin River Delta in California, USA (Delta). The Delta is an inland river delta and estuary which has had MCs and cyanoHABs detected in its waters since 1999 [40]. It provides drinking water to millions of people, irrigates much of the state’s agricultural lands, and provides habitat for many endemic and endangered flora and fauna [41,42]. The Delta is monitored for MCs and other cyanotoxins in the water fraction during the typical bloom months, June through September, yet the benthos and samples taken during months outside typical bloom months have rarely been measured [40,43,44]. The Delta serves as an invaluable water source habitat for both humans and plants and animals; therefore, it is important to effectively quantify MCs in the environment. Currently, it is unclear whether MCs are present in the Delta before or after typical bloom months and whether MCs are present in the benthic environment of the Delta. We hypothesized that (1) MCs are present in the Delta in months outside typical blooms months (hereafter ‘non-typical bloom months’), and that (2) MCs are more prevalent in the benthos than the water fraction, the benthos being represented as sediment and clam tissue in our study. To address these hypotheses, this study optimized an analytical method to detect and quantify both bound and unbound MCs and subsequently quantified MCs in sediment, clam tissue, and water samples collected from sites throughout the Delta during two non-typical bloom months, May and October 2017.

## 2. Results

### 2.1. Optimization of Total MC Quantification (Bound and Unbound)

#### 2.1.1. Hexane Extraction

Employment of a hexane extraction of lipids increased MMPB yield from 56% to 60% for sediment samples and 39% to 56% for clam samples when compared to samples analyzed without hexane lipid extraction. In addition to improved product yield, lipid extraction using hexane decreased the thickness of the undefined third layer when removing MMPB methyl esters from acidified methanol and streamlined extraction process.

#### 2.1.2. Oxidant Concentration

**Figure 2:**
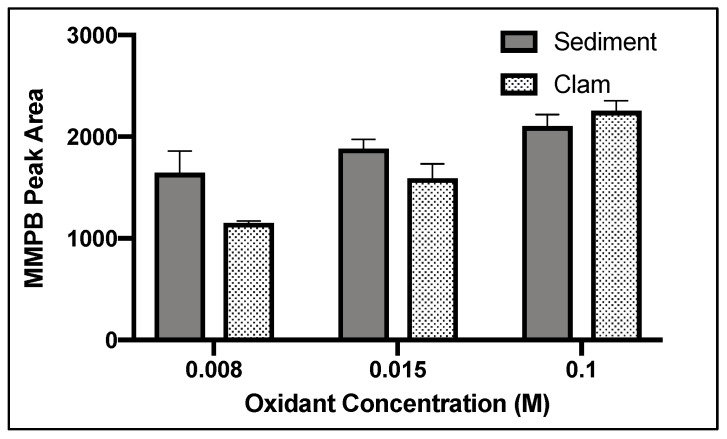
MMPB yield from MC-LR spiked blank sediment and clam samples using three concentrations of oxidant solution for three hours. Each bar represents an average of three samples. Error bars are standard deviations.

Among the concentrations tested, 0.1 M (a saturated solution) returned the highest yield of MMPB produced from MC-LR (specific congener of MC with L-leucine and L-arginine in the variable amino acid positions of the toxin structure) oxidation in soil and clam tissue (Figure 2).

#### 2.1.3. Oxidation Time

**Figure 3:**
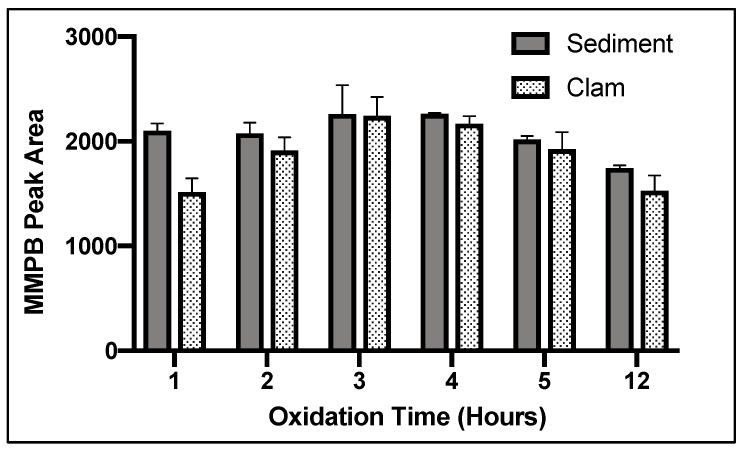
MMPB yield from MC-LR spiked blank sediment and clam tissue matrices when exposed to 0.1 M oxidation solution for 1, 2, 3, 4, 5, and 12 h. Each bar represents an average of three samples. Error bars are standard deviations.

For both clam and sediment, 3 h of oxidation returned the highest product yield (Figure 3).

#### 2.1.4. Solid-Phase Extraction (SPE) 

**Figure 4:**
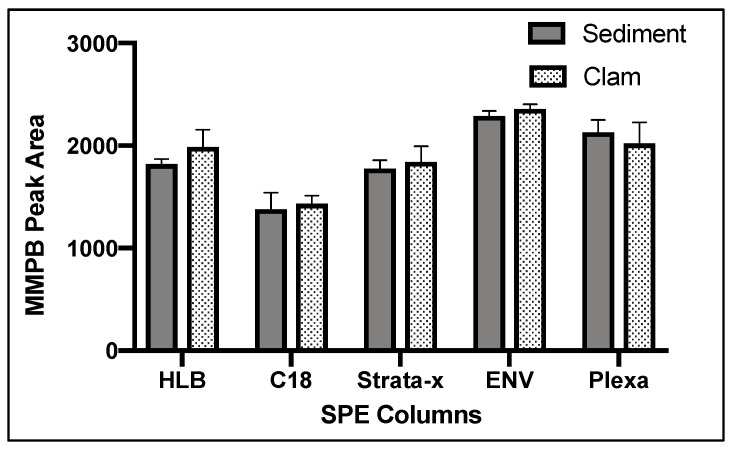
MMPB yield from MC-LR spiked sediment and clam tissue matrices testing five different Solid-Phase Extraction (SPE) columns. Each bar represents an average of three samples. Error bars are standard deviations.

Of five SPE cartridge types tested, ENV returned the highest concentration of product (Figure 4). 

#### 2.1.5. SPE Washes

**Figure 5:**
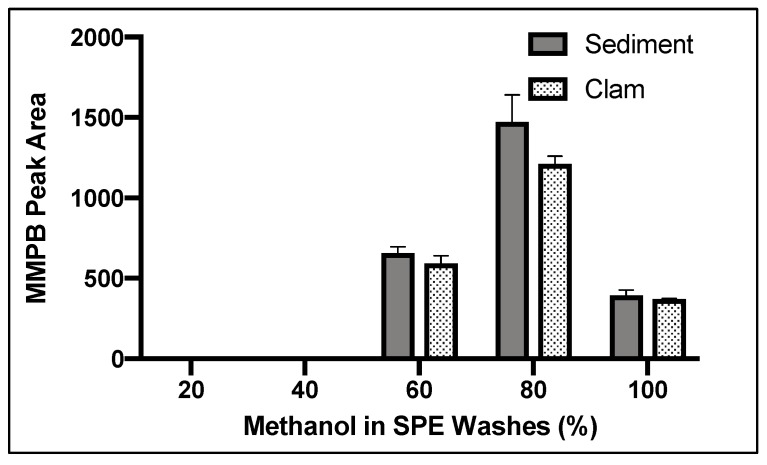
Yield of MMPB with increasing concentrations of methanol in aqueous solution off the ENV SPE columns. Each bar represents an average of three samples. Error bars are standard deviation.

The MMPB product did not begin to elute until the 60% methanol wash and continued to elute from the column until 100% (Figure 5). We concluded that 20% methanol was an efficient wash to remove unwanted contaminants from the sample. The 20% methanol wash was chosen over a 40% methanol wash to be conservative. We concluded that 100% methanol solution was ideal for maximum product elution since product was still eluting off the column with 100% methanol solution after washing with 60% and 80% methanol solution (Figure 5).

### 2.2. Method Validation

The finalized method was validated by spiking blank sediment and clam matrices with 50, 100, and 500 ng of MC-LR per g of dry weight (DW) sample (n = 7 for each matrix–concentration combination). Percent relative standard deviation in each validation concentration spike was 15% or less (Table 1).

**Figure 6:**
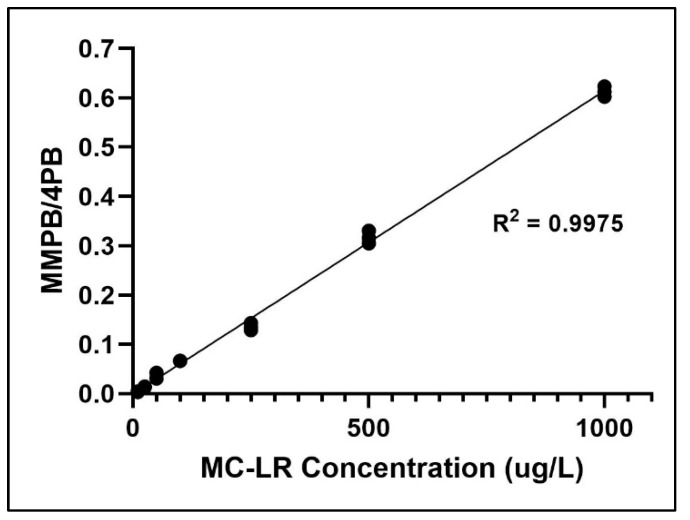
Calibration curve of MC-LR concentrations versus MMPB to 4-phenylbutryic acid (4PB) ratio.

Limit of detection (LOD) was determined by the MMPB standard concentration which was above three times the signal to noise ratio (S/N>3), while limit of quantification above was ten times S/N (S/N>10). LOD/LOQ values of samples were calculated via Mass Hunter Quantitative Analysis Workstation Software. LOD/LOQ for soil and clam tissue was determined to be 10 ng (gDW) ^−1^/50 ng (gDW) ^−1^ and 15 ng (gDW) ^−1^/63 ng (gDW) ^−1^, respectively. Limit of linearity (LOL) was 10 ng (gDW) ^−1^ to 10 mg (gDW) ^−1^. Specificity of MMPB analysis was demonstrated by matching MMPB found in environmental samples to the mass spectra and retention time of MMPB standard and MMPB derived from MC-LR standard. Method blanks were void of qualifier and quantifier ions at the retention times used for quantification of both MMPB and 4-phenylbutryic acid (4PB). The calibration curve for MMPB demonstrates linearity (Figure 6).

### 2.3. Loss of Product

When calculating product yield during the optimization steps, we found that maximum percent recovery was ~50% at best. Each step of the extraction was evaluated for product loss and it was discovered that the major loss was occurring from conversion of MC-LR to MMPB (Table 2).

### 2.4. Optimization of Unbound MC Quantification 

Unbound MCs were extracted from sediment and an enzyme-linked immunosorbent assay was applied. However, the test returned false positives and unreliable calibration curves. Therefore, a method to analyze unbound MCs via the MMPB was optimized. 

#### 2.4.1. Oxidant Concentration

**Figure 7:**
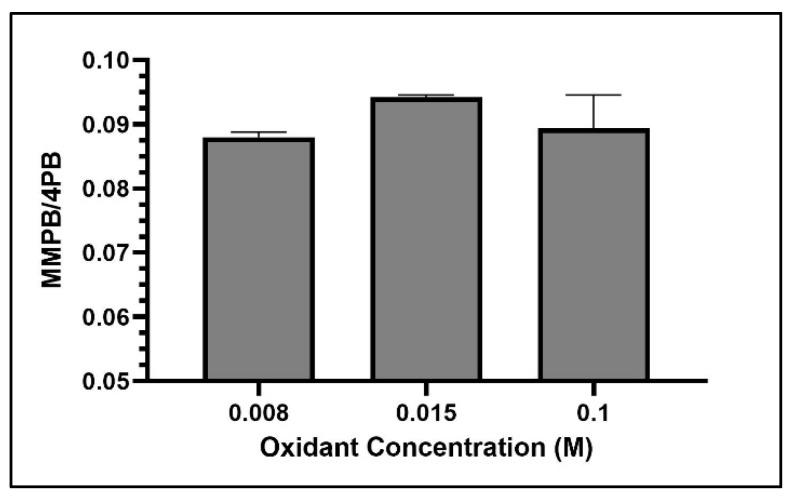
MMPB yield from MC-LR spiked water using three concentrations of oxidant solution. Each bar represents an average of three samples. Error bars are standard deviations.

Among the concentrations tested, 0.015 M returned the highest yield of MMPB produced from MC-LR oxidation (Figure 7). 

#### 2.4.2. Oxidation Time 

**Figure 8:**
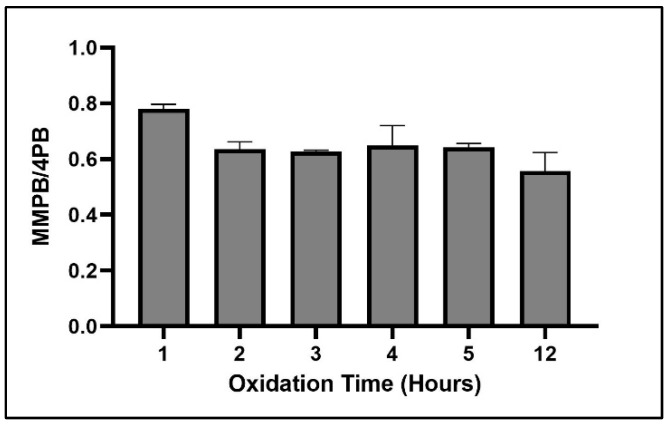
MMPB yield from MC-LR spiked water when exposed to 0.015 M oxidation solution for the above range of oxidation times. Each bar represents an average of three samples. Error bars are standard deviations.

The shortest oxidation time tested (1 h) resulted in the highest return of MMPB (Figure 8).

#### 2.4.3. SPE

**Figure 9:**
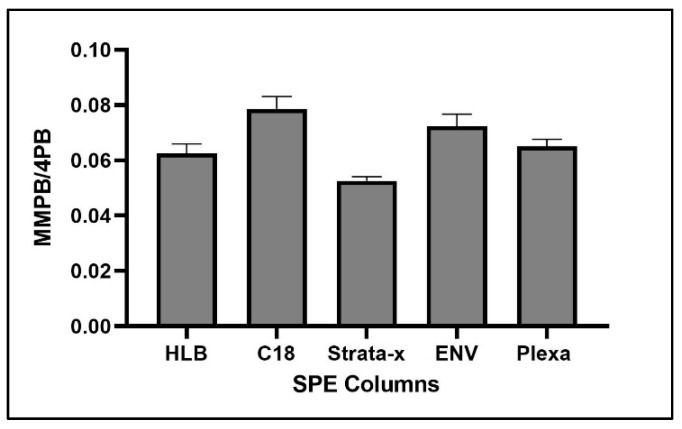
MMPB yield from MC-LR spiked water using five different SPE columns. Each bar represents an average of three samples. Error bars are standard deviations.

For optimization of SPE, C18 returned the highest concentration of product (Figure 9). 

### 2.5. Bound and Unbound Time Series

In order to determine what fraction of MCs became bound over time and to test the extraction efficiency of the bound and unbound MCs methods, a bound and unbound time series experiment was performed. MC-LR was spiked into blank sediment samples, exposed for increasing increments of time, solvent extracted for unbound MCs, and the remaining sediment was analyzed for bound MCs. Bound and unbound MCs time series indicated that MCs become increasingly bound as exposure to matrix increases. In total, 57% of spiked MCs became bound after 72 h of exposure. Between the bound and unbound analysis, 95–113% of spiked MCs were recovered. 

### 2.6. MC Quantification in Environmental Samples 

Total MC concentrations in sediment samples ranged from 59.3 to 161.5 ng (gDW) ^−1^. The majority of MC-positive sediment samples (6/8 or 75%) were collected from sites on the San Joaquin River. Looking at region-specific breakdowns, 6/7 or 85% of sites on the San Joaquin River were found to be positive for MCs, and 2/13 or 15% of sites excluding the San Joaquin River tested positive for MCs. Total MC concentrations in clam samples were below LOD. All the water samples measured during the sampling events via PPIA had MC concentrations below the method’s limit of detection (0.25 µg/L).

## 3. Discussion

### 3.1. Optimization of Total MC Quantification

During optimization, the first major issue we encountered was the presence of what we believed to be lipids in the sediment and clam samples. This issue mainly occurred during the esterification step with the liquid-luqid extraction of the product. The methylated lipids caused the aqueous and hexane layer to separate slower and created an emulsion layer between the aqueous and hexane layer. The GC–MS spectra of the product revealed multiple compounds which matched the spectra of lipid methyl esters when searched against the National Institute of Standards and Technology Spectral Library for GC–MS (EI). The need to remove organic matter from sediment samples for effective MC extraction has been mentioned in prior publications. For example, when analyzing total MCs in lake sediment, Wu et al. reported that samples with more organic content had reduced yield of MMPB [27]. The authors suggested a pretreatment to reduce the interference of organic material with the oxidation of MCs, especially in sediment samples with high organic material. A research summary by Sanan and Lazorchak mentioned compromised sample quality when performing liquid–liquid extraction caused by interferences with fats and oils when extracting total MCs from fish tissue [45]. They reported that this interference had no impact on MMPB percent yield. However, their methods analyzed fish tissue and water, not sediment or clam tissue. The methods for extraction of total MCs from sediment and clam tissue presented in this paper improves the yield of total MCs by utilizing oxidant solution and analysis via GC–MS through the incorporation of lipid extraction prior to oxidation. We believe that hexane extraction allowed removal of excess lipids from samples, which may interfere with oxidation. When lipid extraction was incorporated into the method to quantify total MCs, product yield in sediment and clam samples was improved, less potassium permanganate was required for oxidation, the methylated lipid layer was reduced during hexane extraction, and the time required for extraction was reduced. At first, MTBE was utilized as a lipid extraction solvent. However, higher spikes of MC-LR (500 µg/L) resulted in higher loss (~74%), suggesting a solubility issue. Instead, hexane was used for lipid extraction and increased percent yield across all spike concentrations.

Oxidant concentration, oxidation time, and SPE were important to optimize for MMPB extraction due to the nature of the reactive oxidant solution as well as the complex matrix of sediment and clam tissue. In the literature, there has been concern about possible degradation of MMPB with high oxidant concentrations or long oxidation times [27,46]. However, with a lower concentration of oxidation solution, there may be multiple components in complex matrices competing for oxidation of MCs during the reaction, potentially inhibiting the oxidation of MMPB. Therefore, there must be a balance between providing enough oxidant to oxidize MCs and limiting exposure to avoid degrading the oxidation products. During our optimization of total MC analysis, a saturated solution of potassium permanganate and sodium metaperiodate (0.1 M) returned the highest yield. In contrast, during optimization of unbound MC quantification, 0.015 M returned the highest product yield. This verifies that the matrix is inhibiting the oxidation process. Without the matrix, ~1/10 of the oxidant concentration needed for samples with soil or clam tissue was needed for solvent extracted MCs. During total MC quantification optimization of oxidant time, the yield of MMPB increased between 3 and 4 h and then decreased after 5 h, suggesting that after 4 h the oxidant solution began to degrade the product. During optimization of unbound MCs, this degradation appeared to happen much quicker, after around 2 or 3 h.

The complex matrix of sediment and clam tissue contributed significantly to the optimization of the SPE columns chosen. We originally expected that the HLB or Strata-x would have a higher yield of product due to the specialized sorbent designed to extract moderately polar compounds such as MMPB. However, we found that ENV, a non-polar reverse phase specially designed for the extraction of polar analytes, returned the most product for both the sediment and clam samples (Figure 4). This may be because the specialized sorbents captured more moderately polar compounds, competing with MMPB for the binding locations and allowing product to be lost during load step. Besides the sorbent type, the ENV cartridge had the largest particle size of the cartridges used as well as the lowest sorbent bed mass. Particle size and sorbent bed mass were not optimized in our study but could be important to consider in future studies. For analysis of the unbound MCs, the C18 column returned the highest yield of product. There is less matrix in the unbound MC samples, and therefore the C18 may have been more effective at partitioning MMPB due to larger surface area offered by the smaller particles. 

### 3.2. Product Loss 

**Figure 10:**
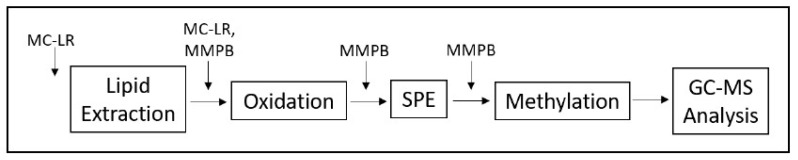
Schematic of spiking experiment to determine product loss.

The overall yield of MMPB from spiking equivalent amounts of MC-LR was 51% across spikes in both sediment and clam matrix. This yield is an improvement on the yields reported by previous studies which used the MMPB method, including a 39% yield of MMPB from MC-LR spiked into dolphin tissue and a 37% yield from fetal bovine serum [47,48]. The relatively low yield of MMPB in complex matrices appears to be a characteristic of the extraction and may be due to the retention of MCs in the matrix. All environmental samples in this study were run with a matrix matched calibration curve to account for such losses. Based on our experiment to determine where the product loss was occurring along the extraction, it appears that the most significant loss occurred when MC-LR was cleaved during oxidation to yield MMPB in both matrices (Table 2, Figure 10). This loss may be due to more than one product being produced from oxidation of MC-LR. 

### 3.3. Time Series of Bound and Unbound MC Fractions

**Figure 11:**
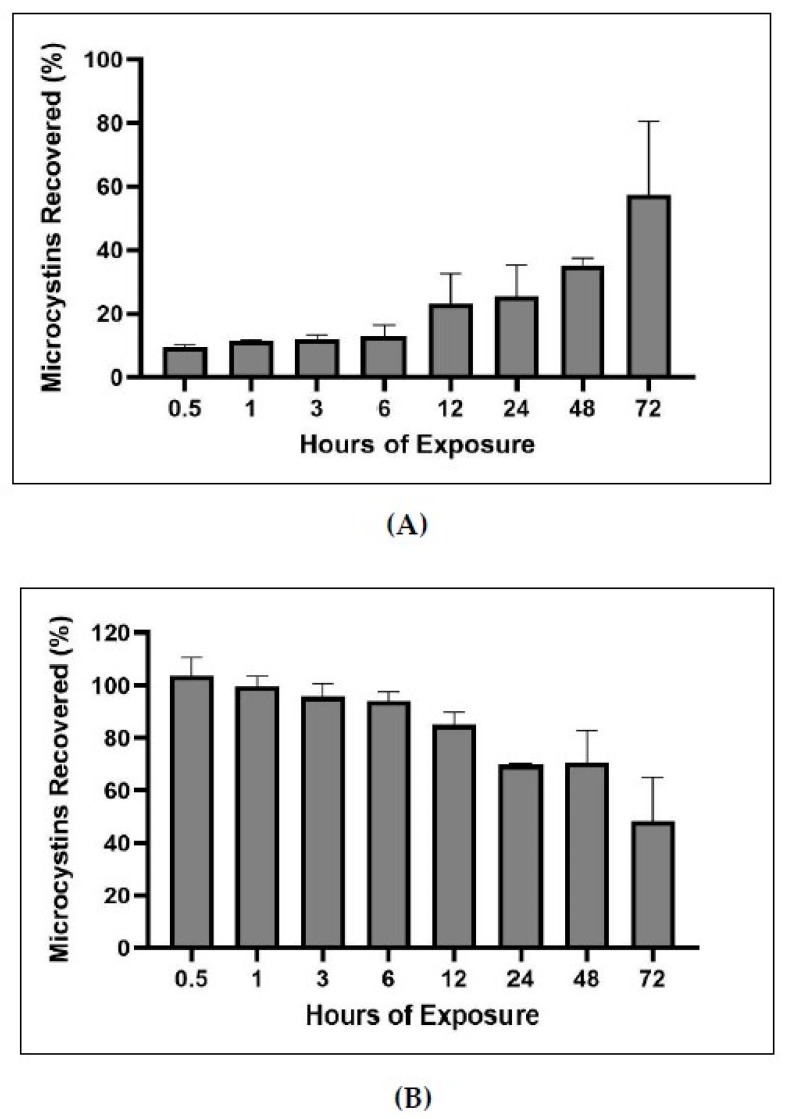
Percentage of MC-LR spike recovered from bound (**A**) and unbound (**B**) MC analysis post exposure of MCs to sediment matrix for 0.5–72 h (n = 3). Note: x-axes not to scale.

The times series of bound and unbound MCs demonstrated that the percentage of bound MCs increases with increased time of exposure to sediment (Figure 11). Further, the experiment demonstrates the utility of the bound and unbound extractions, as the methods recovered nearly all of the MCs spiked in the sediment (95–113%). Craig et al. conducted a time course of covalent adduct formation of MCs binding to protein phosphatases (PPases) in which the PPase-toxin complex was separated from the free PPase by C18 reverse-phase liquid chromatography [15]. They found that within ten minutes of incubation, less than 0.1 mol/mol of MCs to phosphatase complex could be measured, but after 16 h approximately 55% of PPase or 0.55 mol of toxin-PPase complex per mol of PPase was detected. Our experiment shows a similar trend of lower covalently bound MCs detected at short exposure times (less than one hour) versus higher covalently bound MCs detected during longer exposure time (multiple hours; Figure 11). 

### 3.4. MC Detection

MCs were detected in the sediment of the Delta and the concentrations (59.3–161.5 ng gDW ^−1^) fell within typical ranges of MCs detected in similar environments globally (Table 3). MCs in sediments collected from Ariake Bay off the west coast of Japan had a median concentration of carbon-based MC content of 87 ng gDW ^−1^ [49]. Lake Taihu in China had sediments with MC concentrations ranging from 20.4 to 168.1 ng gDW ^−1^ [50]. However, sediment core samples taken from Baptiste Lake in Alberta, Canada revealed concentrations of MCs of over 3000 ng gDW ^−1^ in the top 1–1.5 cm [51], well above the range observed in our study. The MC-positive sediment samples in our study supports our hypothesis that MCs are present in the Delta during non-typical bloom months in the sediment. Due to lack of published exposure studies of MCs in sediment, it is difficult to draw a strong correlation between the levels of MCs detected in sediment and the potential risk to the environment. However, when pumpkinseed sunfish, *Lepomis gibbosus*, were fed phytoplankton with accumulated MCs at a concentration of 50 µg MC gDW ^−1^ for nine days, the fish accumulated MCs in their liver and muscle tissue at concentrations which remained constant even over a two week fasted depuration period [34]. An indirect way to consider these sediment concentrations in water would be to compare parts of toxin per part of matrix. In this case our range of 59.3–161.5 ng g(sediment) ^−1^ could approximately translate to 59.3–161.5 ng mL(water) ^−1^. In an immersion study of MC-LR with zebrafish embryos at 200 ng/L (a concentration approximately 1000 times more dilute than our most concentrated sediment sample when taking into consideration the density of water to compare parts of toxin per parts of matrix) hatchability, heart rate, and glutathione-S-transferase levels were reduced implying bioaccumulation and adverse effects on early development stages [52]. Another study exposed reproducing zebrafish to 25 µg/L for 60 days and demonstrated that MC-LR could be transferred to offspring, inducing developmental neurotoxicity in the larvae [53]. Therefore, at the concentrations found in the sediment, there is potential to adversely affect organisms that live or reproduce on or in the sediment.

**Figure 12:**
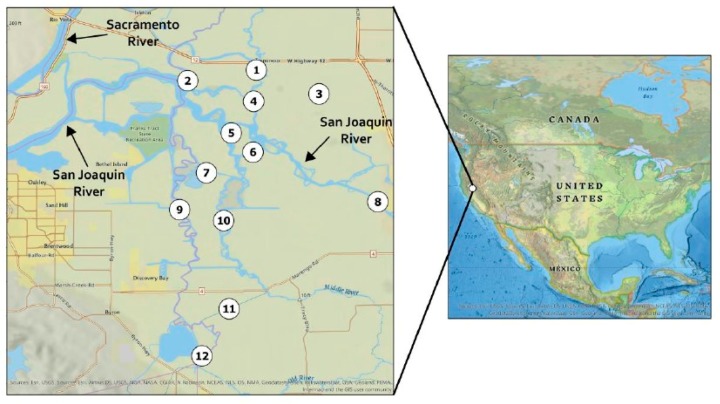
Map of sampling sites in the Sacramento–San Joaquin Delta.

Among the positive sediment samples, 85% of sites on the San Joaquin River were positive for MCs, compared to 15% of the other sites in our study (Table 3, Figure 12). This is consistent with previous work, which showed that the San Joaquin River had higher concentrations of *Microcystis* and higher concentrations of MCs in the algal fraction compared to surrounding regions of the Delta [40,43,54]. Toxin producing *Microcystis* was also detected at multiple sampling sites in the San Joaquin River [55]. The San Joaquin River water quality is more conducive to *Microcystis* blooms, with higher summertime temperatures and nutrient concentrations than surrounding regions. Thus, it is consistent that the San Joaquin River sites exhibited more incidences of MC-positive sediment samples than other sites in the Delta. 

There could be multiple explanations for the higher concentration of MCs in the sediment fraction versus that in water or clam tissue samples. The sediment may provide an ideal environment for growth of MC-producing cyanobacteria. Sediment is often composed of nutrient-rich organic material as well as minerals deposited from surrounding rock formations. For example, Orihel et al. demonstrated that nutrient release from lake sediments stimulated growth of a toxigenic strain of *Microcystis*, which in turn increased the concentrations of MCs [56]. MCs in sediments may also originate from multiple processes including growth or lysis of benthic cyanobacteria cells, adsorption of dissolved MCs to the sediment, and MCs bound in animal tissue or fecal matter post ingestion of MCs by benthic organisms [50]. The non-detects of MCs in the water in our study were likely due to the season we sampled. Colder water temperatures (an average of ~16 °C) during May and October in the Delta, are not an ideal environment for MC-producing cyanobacteria such as the genus *Microcystis*. Though some *Microcystis* strains are more tolerant of low temperatures, in general, *Microcystis* species reduce their metabolic rates and have reduced growth at water temperatures below 15 °C [57,58]. *Microcystis* are also known to sink to the sediment surface during colder months and become dormant until the water becomes warmer, potentially increasing sediment MC concentration [59,60]. The movement of MCs from the water to the sediment fraction could also explain why the clams had MC levels below LOD. Clams are filter feeders; if the water fraction is without MCs clams should not accumulate them. Although we did not find MCs in clam tissue sampled in our study (contrary to our hypothesis), it remains important to test clams for MCs as they are an important food source for humans (particularly in Asian fisheries) as well as species of conservation concern such as sturgeon and diving birds [61,62,63,64]. Moreover, we did not quantify MCs in clam tissue during a bloom, when concentrations would likely be higher.

### 3.5. Contribution to Current Understanding of MC Detection and Distribution

Optimization of total MC quantification uncovered a lipid extraction step that improved workflow and yield of MMPB. The time series experiment comparing percentages of bound and unbound MCs provided additional information on the binding capacity of MCs as the exposure time to sediment increases. Product loss determination at each step of total MC quantification revealed that the major loss of product was coming from the conversion of MC-LR to MMPB. These method improvements may aid in future analysis of MC quantification in complex matrices. The environmental analysis of both the bound and unbound forms of MCs in sediment and clam tissues is the first of its kind in the Delta. These data provide new understanding of the distribution of MCs within the Delta, and how MCs partition in the environment. Our study demonstrates that MCs may still be abundant in the sediment even when water collected from the same locations test negative for the toxins. Thus, it is beneficial to include benthic samples for MC monitoring. Our data also highlight the importance of considering the season at which to analyze MCs. Historically, cyanoHABs take place during the warm summer months, typically between June and September in the northern hemisphere. In our study, MCs were measured and detected in samples collected during non-typical bloom months.

## 4. Materials and Methods 

### 4.1. Materials and Standards

#### 4.1.1. Materials 

The reagents and solvents were of analytical or chromatographic grade. Purified microcystin-LR (MC-LR) was purchased from Cayman Chemicals (Ann Abor, USA). Erythro-2-methyl-3-methoxy-4-phenylbutyric acid (MMPB) was purchased from TCI America (Portland, USA) and 4-phenylbutryic acid (4PB) was purchased from Sigma-Aldrich (St. Louis, MO). Potassium permanganate, potassium bicarbonate, sodium metaperiodate, and sodium carbonate were purchased from MilliporeSigma (Milwaukee, USA). Sodium metabisulfite was purchased from Fisher Scientific (Chicago, USA). Cartridges used for SPE included ENV-Bond Elut (Agilent), 100 mg sorbent mass, 125 µM particle size, 3 mL loading volume; Plexa-Bond Elut (Agilent), 200 mg, 40 µM, 3 mL; C18 (Agilent), 200 mg, 40 µM, 3 mL; HLB (Oasis), 200 mg, 60 µM, 6 mL; and Strata-x (Phenomenex), 200 mg, 33 µM, 6 mL.

#### 4.1.2. Standards

MC-LR is the most abundant congener and considered to be the most toxic. Therefore, MC-LR standards were used to quantify total MC concentration and to validate the extraction method [65]. MC-LR standards were prepared by reconstituting lyophilized MC-LR in methanol and preparing dilutions of 1000, 500, 250, 100, 50, and 25 µg/L in methanol. Sediment and clam matrix used for method optimization and validation were obtained from a local creek where no known harmful cyanobacteria blooms have occurred (Putah Creek). Blank matrix was analyzed to ensure matrix was void of MCs. MC standards were spiked into blank matrix and were subjected to oxidation, extraction, and methylation (see Section 4.2.5). MMPB was dissolved in methanol and prepared via serial dilution to concentrations 1000, 500, 250, 100, 50, and 25 µg/L with methanol. The 4-phenylbutyric acid was used as an internal standard. 

### 4.2. Methods

#### 4.2.1. Site Description

The Sacramento–San Joaquin Delta is an inland delta covering 2990 km^2^ with 1100 km of waterways formed bounded by the Sacramento River in the north and the San Joaquin River in the south [44]. *Microcystis* blooms have occurred annually in the Delta since their first detection in 1999 [40,54]. The blooms typically occur over a four-month period, with their highest concentrations occurring during August and September; and on average, approximately 20% of the cells during *Microcystis* blooms in the Delta contain MCs [40,54,66]. Average MC concentrations between wet and dry years in the Delta usually range from approximately 0.01 to 0.06 µg/L; however, during a recent major drought year (2014), average MC concentrations increased approximately 11–64 times the typical detection range with the highest measured value at 32.9 µg/L [44]. The World Health Organization drinking water and recreational water guidelines for MCs are 1 and 20 µg/L, respectfully. 

#### 4.2.2. Field Sampling

Field sampling was performed at twelve sampling stations throughout the Sacramento–San Joaquin Delta and one field control (Figure 12) during May 10th and 11th and October 2nd and 3rd 2017. Sites 2, 5, 6, and 8 are part of the San Joaquin River system while the remaining sites are more influenced by other water systems. Sediment (n = 20), clam (n = 10), and water (n = 21) samples were collected (Table 4) and stored at 4 °C until delivery to Aquatic Health Program (AHP) at University of California, Davis. Sediment and clam samples were collected via a Ponar grab sampler, which collected the top seven to fifteen centimeters of sediment. Clams were extracted from sediment and stored at 4 °C during transportation from Delta. Each water sample was collected from 0.5 to 1 m below water surface. Samples were stored on boat and during transportation to AHP at 4 °C. Once at AHP, water samples were stored at -20 °C until analysis, while sediment and clams were stored at -80 °C until analysis.

#### 4.2.3. Analysis of MCs in Water 

The unbound MC concentrations in water samples were measured by protein phosphatase inhibition assay (PPIA) for MCs using commercially available kits (Abraxis, Warminser, PA, USA) and analyzed according to the instructions of the manufacturer. Extracellular MCs were measured from unfiltered water samples. For intracellular MCs, water samples were filtered with glass fiber filters (934-AH borosilicate filters; pore size 1.5 µm) to obtain cyanobacteria cells which were then lysed with 80% methanol in water. Summations of MCs in algal and dissolved fractions were used to obtain concentration of unbound MCs in water samples.

#### 4.2.4. Treatment of Sediment and Clam Tissue Samples 

Sediment samples were dried in a vacuum oven at 60 °C and 20 mmHg and then sifted through a 1 mm^2^ sieve. Clam tissues were removed from their shells, frozen in liquid nitrogen and then freeze dried to remove water content. After drying, 1 g of sediment and 0.1 g of clam tissue were used for analysis. 

#### 4.2.5. Quantification of Total MCs (Bound and Unbound) 

*Optimization.* The quantification of total MCs was executed by spiking MC-LR into blank sediment or clam tissue and optimizing five parameters in the extraction: lipid extraction (hexane and MTBE solvents), oxidant solutions (0.008, 0.015, and 0.1 M), oxidation time (1, 2, 3, 4, 5, and 12 h), SPE cartridges (HLB, C18, Strata-x, ENV, and Plexa), and SPE washes (20, 40, 60, 80, and 100% methanol). Lipid extraction optimization was performed with 0.1 M oxidant solution for three hours of oxidation and product was extracted with an ENV column. Oxidant concentration optimization was performed for three hours of oxidation and product was extracted with a C18 column. Oxidation time optimization was performed with 0.1 M oxidant solution and product was extracted with a C18 column. SPE column optimization was performed with 0.1 M oxidant solution for three hours of oxidation. All SPE cartridges were conditioned with methanol and water, eluted with 20% methanol, and product eluted with methanol on a vacuum manifold. For SPE wash optimization, three hours of oxidation with 0.1 M oxidant solution was used with an ENV column. Resulting MMPB was eluted from the column via sequential 2 mL aqueous washes with increasing amounts of methanol (0%, 20%, 40%, 60%, 80%, and 100%) and each fraction was analyzed for MMPB concentration. The level of each parameter that returned the highest yield of product was incorporated into the final protocol described below. This protocol was used to (1) quantify product loss for each step (4.2.7), (2) quantify bound MCs in the bound and unbound MC time series (4.2.8), and (3) total MC quantification of field samples (4.2.9).

*Lipid extraction.* Total MCs were quantified via an extraction process from soil and clam matrices via an adapted oxidation method with potassium permanganate and sodium metaperiodate [27]. Hexane extraction was employed to remove lipids [67]. Samples and methanol (2 mL) were mixed in a polypropylene tube. The mixture was sonicated on ice for 10 min to disrupt matrix. Hexane (5 mL) was added and solution was shaken at room temperature for 1 h. Water was added (2 mL) to separate organic and aqueous layers. The sample was then centrifuged at 5000× *g* for 5 min and the hexane layer was removed. The remaining solution was dried under a stream of nitrogen at 35 °C for 1 h to remove any residual hexane. 

*Oxidation.* The samples were subjected to oxidation to cleave the Adda moiety from bound MCs, resulting in liberation of 3-methyl-2-methoxy-phenylbutryic acid (MMPB). MMPB concentration was used as an indirect detection of covalently bound MCs as well as unbound MCs (total MCs). For the oxidation step, an aqueous solution of 0.1 M potassium permanganate and sodium metaperiodate had pH adjusted to 9 with equal molarity of potassium bicarbonate and sodium carbonate. Oxidant solution (10 mL) was added to sediment sample and shaken at room temperature for three hours. Excess potassium permanganate was added as needed throughout the reaction to maintain purple color throughout the reaction, a visual indication of permanganate’s oxidizing capacity. After oxidation, reaction was quenched with 1–1.5 g of sodium bisulfite. The solution was adjusted to pH 2 with 10% sulfuric acid to ensure that the resulting MMPB product was protonated. The sample was centrifuged at 5000× *g* for 5 min. 

*SPE.* ENV cartridge was conditioned with methanol and MilliQ water. The solution was then passed through an ENV SPE cartridge, washed with MilliQ water to remove salts (6 mL) and 20% methanol to remove contaminants (6 mL), and was then eluted with 100% methanol (2 mL). 

*Esterification.* MMPB eluent was esterified with hydrochloric methanol (5%, 1 mL) and heated at 70 °C for 1 h. Hexane (2 mL) and NaCl saturated water (2 mL) were added to esterified MMPB and solution was shaken. Once settled the hexane layer was extracted three times, combined, concentrated to a volume of 1 mL with a stream of nitrogen in a 25 °C water bath. The sample was then subjected to GC–MS analysis. 

#### 4.2.6. Quantification of Unbound MCs 

*Removal of MCs from sediment.* Analysis of unbound MCs was determined using an altered protocol from Wu et al. [27]. Dried sediment samples (1 g) were extracted three times with sequential solvent extraction with 50% (v/v) methanol (20 mL) and 0.1M EDTA-0.1M Na_4_PO_7_ (pH~3) (20 mL). For each extraction, sample and solvent slurry were sonicated for 10 min at 4 °C in a water bath. After sonication, the sample was centrifuged at 5000× *g* for 5 min and aqueous extracts were combined. Extracts were then passed through a C18 SPE cartridge, washed with MilliQ water (6 mL), washed with 20% methanol (6 mL), and eluted with 100% methanol (2 mL). This extraction removed the unbound MCs from the sediment samples. 

*Optimization of unbound MC quantification.* The quantification of unbound MCs was executed by spiking MC-LR into MillQ water and optimizing three parameters in the extraction: oxidant solutions (0.008, 0.015, and 0.1 M), oxidation time (1, 2, 3, 4, 5, and 12 h), SPE cartridges (HLB, C18, Strata-x, ENV, and Plexa). Oxidant concentration optimization was performed for three hours of oxidation and product was extracted with a C18 column. Oxidation time optimization was performed with 0.015 M oxidant solution and product was extracted with a C18 column. SPE column optimization was performed with 0.015 M oxidant solution for one hour of oxidation. The level of each parameter that returned the highest yield of product was incorporated into the final protocol described below. This protocol was used to (1) quantify unbound MCs in the bound and unbound MC time series and (2) total MC quantification of field samples (4.2.9).

*Analysis of unbound MCs.* Extracted unbound MCs were subjected to oxidation with 0.015 M potassium permanganate and sodium metaperiodate (10 mL) with pH adjusted to 9 with equal molarity of potassium bicarbonate and sodium carbonate and shaken for 1 h at room temperature. The solution was quenched, and the pH was brought down to ~2 in the same manner as the quantification of total MCs. The solution was then passed through a C18 SPE that had been conditioned with methanol and MilliQ water, washed with MilliQ water (6 mL), washed with 20% methanol (6 mL), and product was eluted with 100% methanol (2 mL). The esterification process was followed from quantification of total MCs, and samples were subjected to GC–MS analysis.

#### 4.2.7. GC–MS Analysis

Samples were run on an Agilent GC System 7890B (Agilent, Santa Clara, CA, USA) with an MSD 5977A. A DB-5MS (30m x 0.25 mm ID x 0.25 µm) column was used to separate compounds. The temperature program started at 50 °C, with a hold time of 1 min, followed by 15 °C/min ramp until 157 °C, 8 °C/min ramp until 185 °C, and finally 15 °C/min ramp until 280 °C with a 5 min hold time. Methylated MMPB was quantified using the ion 131 *m/z* and verified with the qualifier ions 135 *m/z* and 191 *m/z*. The 4-phenylbutyric acid (4PB) was used as a surrogate and its methylated form was quantified using the ion 104 *m/z* and verified with the qualifier ions 91 and 74 *m/z*. The 4PB was found to degrade under high oxidant concentration, and therefore 4PB was added into samples post oxidation. MMPB and 4PB were quantified by use of a linear standard curve using commercially available standards. 

#### 4.2.8. Loss of Product

To determine where loss of MC-LR and MMPB was occurring during the reaction, we spiked MC-LR and MMPB at the molar equivalent to 1 g of dried blank sediment and 0.1 g of dried blank clam tissue at different points throughout the extraction and analyzed loss at each step of the optimized method (Figure 10).

#### 4.2.9. Bound and Unbound MCs Time Series

A time series experiment was executed to determine what fraction of MCs became bound over time and to test the extraction efficiency of the bound and unbound MCs methods. MC-LR was spiked at 1000 µg/L into 1 g of dried blank sediment, vortexed to ensure equal distribution of MC-LR in sediment sample, incubated at room temperature, and extracted after 0.5, 1, 2, 3, 6, 12, 24, 48, and 72 h post exposure to sediment. Each time point had individual samples (n = 3). MC-LR spiked sediment was first extracted for unbound MC quantification and the remaining sediment was extracted for bound MC quantification. 

#### 4.2.10. Analysis of Environmental Samples

Soil and clam samples collected from the Sacramento–San Joaquin Delta were processed and analyzed for total MCs. Samples which tested positive for total MCs were then analyzed for unbound MCs.

## Figures and Tables

**Table 1:** Validation results for sediment and clam tissue matrices for total MC quantification (n = 7). MC-LR spike amounts are followed by MMPB percent yield and relative standard deviation (RSD) of MMPB in each matrix.

Spike (ng gDW ^−1^)	Sediment		Clam Tissue	
	Yield (%)	RSD (%)	Yield (%)	RSD (%)
50	44–53	9	41–56	10
100	50–55	3	44–54	13
500	47–53	4	39–42	3

**Table 2:** Loss of MMPB derived from MC-LR oxidation at each step of total MC quantification in sediment and clam tissue.

Matrix	Spike (µg/L)	Lipid Extraction (%)	MC-LR to MMPB Conversion (%)	Oxidation (%)	SPE (%)	Overall Loss (%)
Sediment	100	3.3	20.6	11.2	13.3	48.5
Sediment	500	5	29.5	7.3	5.5	47.3
Clam	100	4.5	39.5	5.7	5	54.6
Clam	500	4	32.3	11.6	12.9	60.9

**Table 3:** Total and unbound MC concentrations in sediment samples.

Sample Site	Total MC Concentration (ng gDW ^−1^) ± SD		Unbound MC Concentration (ng gDW ^−1^) ± SD	
	**May 2017**	**October 2017**	**May 2017**	**October 2017**
1	<LOD	<LOD	<LOD	<LOD
2	59.3 ± 3.8	<LOD	<LOD	<LOD
3	20.4 ± 4.4 *	<LOD	<LOD	<LOD
4	<LOD	<LOD	<LOD	<LOD
5	161.5 ± 21.6	27.6 ± 5.0 *	<LOD	33.3 ± 0.2 *
6	114.9 ± 4.9	81.8 ± 6.9	<LOD	<LOD
7	<LOD	<LOD	<LOD	<LOD
8	N/A	23.4 ± 6.1 *	<LOD	<LOD
9	<LOD	N/A	<LOD	<LOD
10	40.6 ± 5.2 *	<LOD	<LOD	<LOD
11	N/A	<LOD	<LOD	<LOD
12	<LOD	<LOD	<LOD	<LOD

* Asterisk indicates value higher than LOD but lower than LOQ, and therefore values are estimations.

**Table 4:** List of water, clam, and sediment samples collected.

Collection Date: May 2017					Collection Date: October 2017				
Day Sampled	Sample Site	Water	Clam	Sediment	Day Sampled	Sample Site	Water	Clam	Sediment
May 10	1	x		x	Oct 2	1	x		x
May 10	2	x		x	Oct 2	2	x	x	x
May 10	3	x	x	x	Oct 2	3	x		x
May 10	4	x	x	x	Oct 2	4	x	x	x
May 10	5	x	x	x	Oct 2	5	x		x
May 10	6	x		x	Oct 2	6	x		x
May 10	7	x		x	Oct 3	7			
May 10	8				Oct 2	8	x	x	x
May 10	9	x	x	x	Oct 3	9			
May 10	10	x	x	x	Oct 3	10	x	x	x
May 10	11				Oct 3	11	x	x	x
May 10	12	x		x	Oct 3	12	x		x
May 10	FC *				Oct 2	FC *	x		

* FC = field control. Field control was deionized water stored in 1 L polypropylene bottle and transported to field alongside samples. X = sample collected.
